# Chitosan Derivatives and Their Application in Biomedicine

**DOI:** 10.3390/ijms21020487

**Published:** 2020-01-12

**Authors:** Wenqian Wang, Qiuyu Meng, Qi Li, Jinbao Liu, Mo Zhou, Zheng Jin, Kai Zhao

**Affiliations:** 1 Key Laboratory of Chemical Engineering Process and Technology for High-efficiency Conversion, College of Heilongjiang Province, College of Chemistry Engineering and Materials, Heilongjiang University, Harbin 150080, China; wangwenqian3104@163.com (W.W.); m940584524@163.com (Q.M.); liqi114013@163.com (Q.L.); ljb19970107@163.com (J.L.); 2 Engineering Research Center of Agricultural Microbiology Technology, Ministry of Education, Heilongjiang University, Harbin 150080, China; zhoumo_wk@hotmail.com; 3 Key Laboratory of Microbiology, College of Heilongjiang Province, School of Life Science, Heilongjiang University, Harbin 150080, China

**Keywords:** chitosan derivative, nanoparticles, biomedicine, drug delivery, immunology

## Abstract

Chitosan is a product of the deacetylation of chitin, which is widely found in nature. Chitosan is insoluble in water and most organic solvents, which seriously limits both its application scope and applicable fields. However, chitosan contains active functional groups that are liable to chemical reactions; thus, chitosan derivatives can be obtained through the chemical modification of chitosan. The modification of chitosan has been an important aspect of chitosan research, showing a better solubility, pH-sensitive targeting, an increased number of delivery systems, etc. This review summarizes the modification of chitosan by acylation, carboxylation, alkylation, and quaternization in order to improve the water solubility, pH sensitivity, and the targeting of chitosan derivatives. The applications of chitosan derivatives in the antibacterial, sustained slowly release, targeting, and delivery system fields are also described. Chitosan derivatives will have a large impact and show potential in biomedicine for the development of drugs in future.

## 1. Introduction

With the improvement of living standards, people have been paying more attention to the development of health and medical technology. In recent years, many polymer compounds, extracted from starch, liver sugar, inulin, cellulose, chitin, and alginates, have been widely used in biology, medicine, beauty, healthcare, and other fields [1,2,3]. Here, chitosan is one area of focus. Chitosan is a deacetylated product of chitin, which is an abundant natural resource that features less storage than cellulose [4,5]. Chitosan is a renewable natural alkaline polysaccharide that has no toxicity and no side effects, and it features good moisturizing and adsorption properties. The United States Food and Drug Administration (FDA) has approved that chitosan is safe in the use of foods and drugs.

However, chitosan is insoluble in water and most organic solvents, which limits its applications in various fields. Chitosan derivatives can be obtained by the chemical modification of chitosan-reactive functional groups. Here, the −OH and −NH_2_ active groups on the chitosan molecule are prone to chemical reactions [6,7]. Chemical modification can not only improve the physical and chemical properties of chitosan, it can also retain the unique properties of chitosan and expand the application range of chitosan derivatives. Modified chitosan derivatives have better biocompatibility, bioactivity, biodegradability, and non-toxicity, and they still possess the original bactericidal, antibacterial, anticancer, and antiviral pharmacological effects, including the ability to induce erythrocyte aggregation, promote platelet activation, and activate complement systems other than that of chitosan [8,9,10,11,12,13]. At present, chitosan derivatives have been widely used in both medical materials and biomedicine. With the development of nanotechnology, chitosan derivatives have been prepared as nanomaterials, including nanoparticles, hydrogels, microspheres, and micelles. Chitosan derivatives can be used as targeted delivery vehicles for drugs, as well as adjuvants and delivery carriers for vaccines [14,15,16,17,18,19,20]. Therefore, chitosan derivatives and their nanomaterials can be widely used and expanded upon in terms of the fields of chitosan application [21,22]. The properties of materials determine their applications, so this review focuses on the preparation of chitosan derivatives with excellent solubility, pH sensitivity, targeting, and mucosal adhesion; additionally, this review introduces the application fields of chitosan derivatives in medicine in three aspects, namely as drug carriers, drug materials, and for mucosal immunity. The effects of chemical modification and different material states (nanoparticles, fibers, gels, etc.) of the properties of chitosan derivatives have been researched. We hope that the review can provide some guidance for research on improving the good properties of chitosan and expanding the potential applications of chitosan.

## 2. Modification of Chitosan

**Figure 1:**
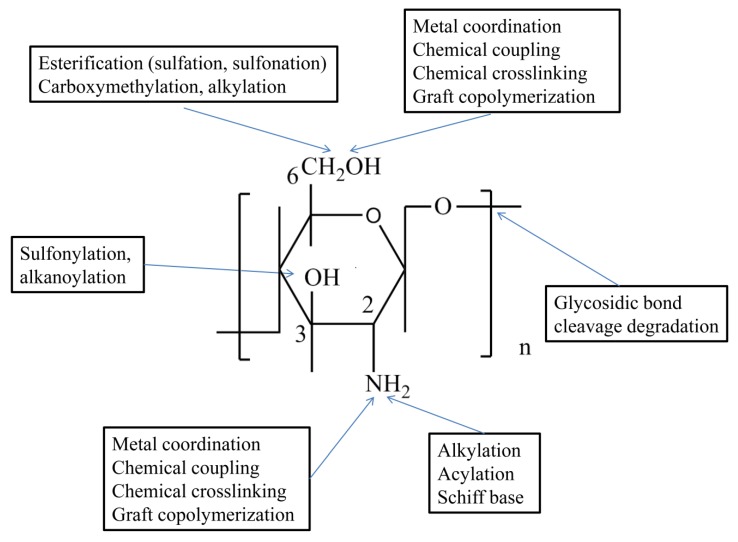
Schematic diagram of chitosan chemical reaction.

Functional groups on the chitosan molecules include C_3_–OH, C_6_–OH, C_2_–NH_2_, and acetyl amino and glycoside bonds [6]. Among them, the acetyl amino bond is as stable as the glycosidic bond, which is not easy to fracture. C_3_–OH belongs to a secondary hydroxyl, it cannot rotate freely, and its steric hindrance is so big that it does not easily react. The active chemical properties of C_6_–OH and C_2_–NH_2_ take advantage of these groups in chitosan molecules to introduce other groups through various kinds of molecular design. The chemical modification of chitosan can improve its physical and chemical properties, as well as expand its applications and relevant research fields [23,24,25]. A schematic diagram of chitosan modification is shown in Figure 1.

### 2.1. Improving the Solubility of Chitosan

Though chitosan has a wide range of applications, its intermolecular and intramolecular hydrogen bonds are highly crystalline, which makes it almost insoluble in water and therefore limits its applications to some extent. Therefore, it is important to improve the water solubility of chitosan. At present, there are generally three considered methods for improving the solubility of chitosan: (1) Chitin deacetylation, where the chitosan, after deacetylation, can only be dissolved in an acidic solution, thus limiting its applications; (2) chemical modification, where a hydrophilic group is introduced on an amino group or a hydroxyl group in a chitosan molecule. At the same time, this method destroys the original hydrogen bond and crystallinity of chitosan; (3) chitosan degrades into a water-soluble product of small molecules under the action of an enzyme, where the molecular weight distribution of chitosan during the degradation is extremely uneven and the product is difficult to separate. Therefore, chemical modification is often used to improve the water solubility of chitosan.

#### 2.1.1. Destruction of Hydrogen Bonding

##### Acylated Modified Chitosan

Acylation modification is the most common modification of chitosan. The acylation of chitosan refers to the reaction of chitosan with a variety of organic acids and derivatives of organic acids (mainly anhydride and acyl chloride), introducing aliphatic or aromatic acyl groups to the molecular chain [26]. The acylation reaction destroys the intramolecular and intermolecular hydrogen bonding of chitosan, which weakens its crystallinity and enhances its water solubility. There are two hydroxyl groups on the molecular chain of chitosan, with one being the primary hydroxyl group of C_6_–OH and the other being the secondary hydroxyl group of C_3_–OH. The primary hydroxyl group is free to rotate in the spatial conformation, with low steric hindrance, while the secondary hydroxyl group is not able to rotate, with high steric hindrance, and the activity of C_2_–NH_2_ is higher than that of the primary hydroxyl group. Therefore, the order of activity of the acylation reaction is C_2_–NH_2_ > C_6_–OH > C_3_–OH [27,28]. An acylation reaction that occurs with C_2_–NH_2_ to form an amide is called N-acylation [29]. An ester is formed by the acylation reaction of C_6_–OH when there is a protective functional group on C_2_–NH_2_, and this is referred to as O-acylation [30].

**Figure 2:**
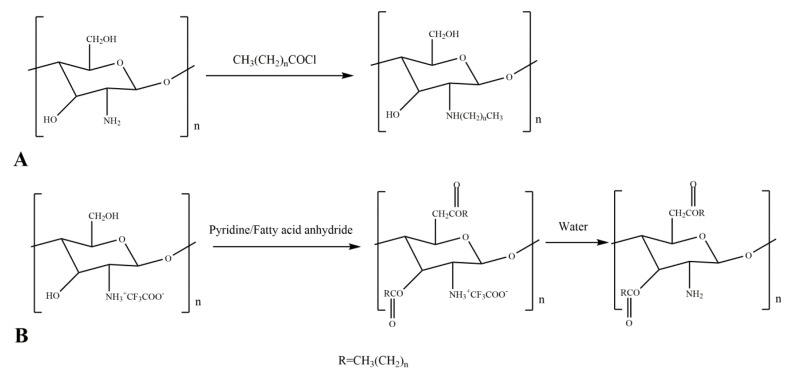
Reaction equations for acylated chitosan derivatives. (**A**) N-acylated chitosan; (**B**) O-acylated chitosan.

N-acylated chitosan derivatives show enhanced biocompatibility, anticoagulability, and blood compatibility. Moreover, N-acylated chitosan derivatives do not cause an inflammatory reaction in the human body, so N-acylated chitosan can be used as a carrier or sustained release agent in pharmaceutical applications [24,29,31,32]. A schematic diagram of the N-acylation reaction of chitosan is shown in Figure 2A.

The solubility of N-acylated chitosan depends on the degree of substitution (DS) and the length of the side chain. Studies have shown that when the DS is less than 50%, the DS is proportional to the solubility; the higher the DS is, the greater the solubility. The length of the side chain is proportional to the crystallinity, and a longer side chain results in a higher crystallinity and lower relative solubility [6,29]. N-acylated chitosan, with high solubility, can be used as a carrier for hydrophobic drugs [33], while N-acylated chitosan, with high crystallinity, can increase fiber toughness and thermal stability, making it suitable for applications such as use in polyvinyl chloride (PVC) fiber film materials [34,35]. Additionally, N-acylated chitosan can also be used as a template additive for bone tissue materials, such as 3D templates for hydroxyapatite [24].

C_6_–OH does not react until C_2_–NH_2_ completely reacts [36]. If only O-acylated chitosan is required, it is necessary to add a solvent to protect the ammonium group, such as trifluoroacetic acid or methanesulfonic acid [37,38,39]. Methanesulfonic acid is both a solvent and a catalyst in the chitosan O-acylation process [37,38]. A schematic diagram of the O-acylation reaction of chitosan is shown in Figure 2B.

O-acylation modification destroys the hydrogen bond structure of chitosan and improves its fat solubility and hydrophobicity. However, the properties of O-acylated chitosan and N-acylated chitosan are also different. O-acylated chitosan is lipid-soluble and can be dissolved in non-polar solvents such as pyridine and chloroform [37], while N-acylated chitosan improves water solubility [29,31]. O-acylated chitosan is commonly used in the films of fibers or polymeric materials to enhance the hydrophobicity and stability of the material [30,38]. N-acylated chitosan can be used as a carrier or a sustained release agent in the delivery of drugs and can also be used as a material additive in biological scaffolds [28,29].

##### Alkylation Modified Chitosan

An alkyl group can be introduced into chitosan, and this leads to the latter having significantly weakened intermolecular hydrogen bonds, leading to an improvement in its solubility [40]. However, the alkyl group is a hydrophobic group. The solubility is lowered when alkyl chain that is too long is introduced, showing that the solubility of a chitosan derivative can be controlled by manipulating the length of an alkyl chain [41,42]. An alkylation reaction is caused by the introduction of an alkyl group to the C_2_–NH_2_, C_6_–OH, or C_3_–OH groups of chitosan to form an alkyl group-containing chitosan derivative. N-alkylation occurs with the alkylation of the C_2_–NH_2_ group in chitosan, and O-alkylation occurs with the alkylation of the C_6_–OH or C_3_–OH groups in chitosan [43,44]. Here, the C_2_–NH_2_ group has strong nucleophilic lone pair electrons, and, thus, N-alkylation is more likely to occur.

**Figure 3:**
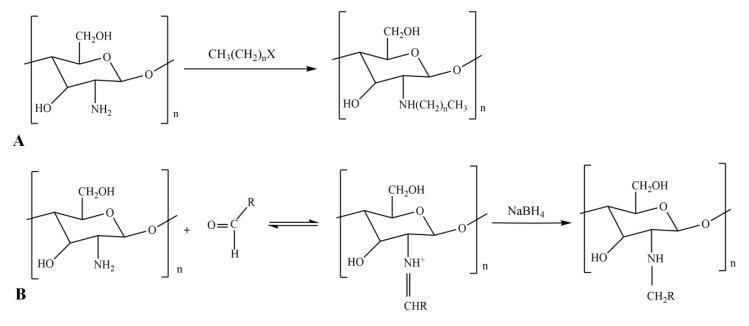
Alkylation chitosan derivative reaction equations. (**A**) Halogenated alkane to prepare N-alkylated chitosan; (**B**) advanced fatty aldehyde prepares N-alkylated chitosan.

N-alkylated chitosan can be prepared from a halogenated alkane. The reaction equation of this process is shown in Figure 3A [45]. The material can also be prepared from higher fatty aldehydes and long chain fatty acyl groups. In this case, the reaction equation is shown in Figure 3B [46,47,48]. Alkylated chitosan can be used to prepare medical gauze due to its coagulation and antibacterial properties [42,47,49], and it can be used to absorb anionic surfactants in water purification engineering due to its positive charge [50].

#### 2.1.2. Introduction of Hydrophilic Group

A hydrophilic group has an atomic group that is soluble in water or that which is readily compatible with water. Common hydrophilic groups include the carboxylic acid group, the quaternary ammonium group, the sulfonic acid group, the phosphoric acid group, amino group, ether bonds composed of an oxygen group, the hydroxyl group, the carboxylate group, and the block polyether group.

##### Carboxylated Chitosan

A carboxylation reaction mainly utilizes glyoxylic acid or chloroalkanoic acid to react with the C_6_–OH or C_2_–NH_2_ groups of chitosan, the product of which is a −COOH group [51]. Carboxylated chitosan has good water solubility and can be dissolved in neutral and alkaline solutions. Carboxylated chitosan also has better thickening, heat preservation, film formation, flocculation, and kneading properties than chitosan. At the same time, carboxylated chitosan has wider applications than chitosan in the industrial, agricultural, medical, health, and biochemical fields [47,50,52,53,54,55,56,57].

**Figure 4:**
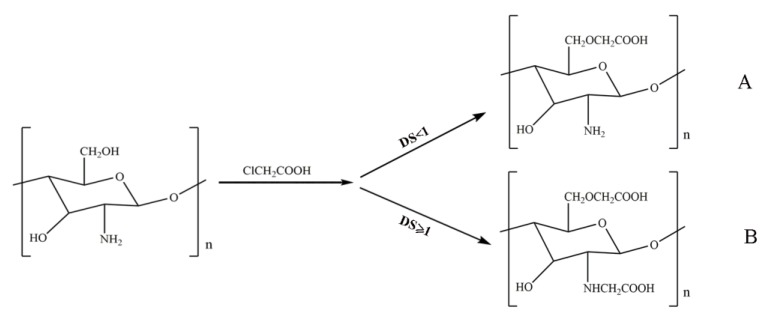
Carboxylated chitosan derivative reaction equation. (**A**) O-carboxymethyl chitosan (degree of substitution (DS) < 1); (**B**) N, O-carboxymethyl chitosan (DS ≥ 1).

Most studies on carboxylated chitosan have concerned carboxymethylation reactions [58,59,60]. The steric hindrance effect on the chitosan C_3_–OH group makes the carboxymethylation of the C_3_–OH group more difficult. Therefore, the carboxylation reaction mostly occurs with the C_6_–OH group. Under an alkaline condition, the activity of the C_6_–OH group on the molecular level of chitosan is greater than that of the C_2_–NH_2_ group. Therefore, when the DS is less than 1, the product is C_6_-*O*-carboxymethyl, and the reaction equation of this is shown in Figure 4A. When the DS is greater than or equal to 1, carboxymethyl substitution occurs simultaneously with the C_6_–OH and C_2_–NH_2_ groups to form N, O-carboxymethyl chitosan [61,62], and the reaction equation of this is shown in Figure 4B.

The property of carboxymethyl chitosan (CMCS) is related to the DS, where the DS depends on the amount of the carboxylating agent and the molecular weight of chitosan (CSMW). The relationship between the CSMW and the DS is that the DS decreases with the increase of the CSMW [63]. CMCS is active in the biomedical and pharmaceutical fields due to its antibacterial properties, which promote wound healing, as well as its lipid-lowering, anti-arteriosclerosis, antiviral, anti-tumor, anti-coagulation, and hypoglycemic effects [51,64].

##### Quaternary Ammonium Chitosan

The quaternary ammonium group is a hydrophilic group and is positively charged. The introduction of a quaternary ammonium salt group not only improves water solubility, it also increases chargeability. The quaternization occurs with C_2_–NH_2_. Quaternization generally occurs via three methods, namely direct quaternary ammonium substitution, N-alkylation, and the epoxy derivative open loop method [65,66,67,68,69]. In recent years, the most commonly used quaternary ammonium salts have been GTA (2,3-epoxypropyl trimethyl ammonium chloride) and CTA (3-chloro-2-hydroxypropyl trimethyl) [15,70].

**Figure 5:**
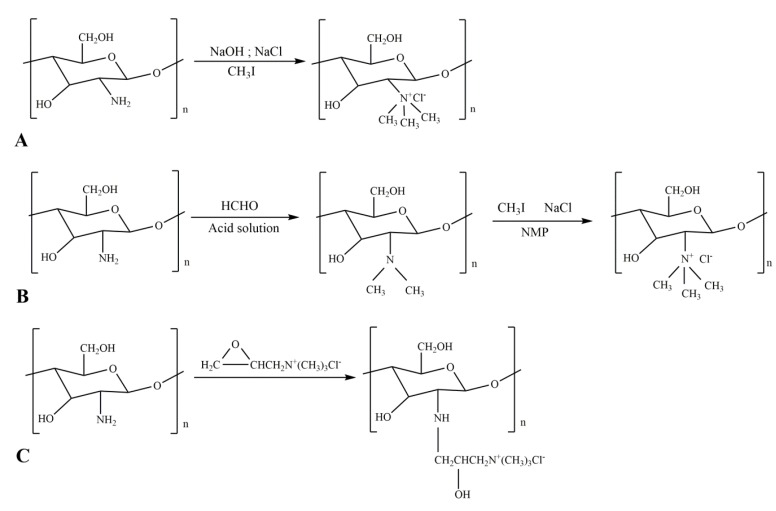
Reaction equations for quaternized chitosan derivatives. (**A**), N,N-trimethyl chitosan (TMC) direct quaternary ammonium salt substitution method; (**B**) TMC N-alkylation; (**C**) chitosan 2,3-epoxypropyl trimethyl ammonium chloride (GTA) ring opening method.

N,N,N-trimethyl chitosan (TMC) is a quaternary ammonium chitosan. TMC can be synthesized by two methods. One method is direct quaternary ammonium substitution (Figure 5A) [71,72], and the other is the N-alkylation method (Figure 5B) [73,74,75]. The epoxy-derivative ring-opening process is the reaction of C_2_-NH_2_ with GTA or CTA under alkaline conditions. The reaction of chitosan and epoxypropyl trimethyl ammonium chloride gives *N*-2-Hydroxypropyl trimethyl ammonium chloride chitosan (N-2-HACC) [76,77]. The reaction equation of this is shown in Figure 5C.

Quaternary ammonium salt increases charging strength and weakens hydrogen bonds, thus increasing water solubility. In addition, a higher DS leads to a better water-solubility and a higher potential [78,79]. Quaternary ammonium chitosan salt also has better antibacterial, biocompatibility, biodegradability, non-toxicity, and biological effects, as well as innate mucoadhesiveness and the ability to penetrate mucus layers and bind to epithelial surfaces. Therefore, it is widely used in medicine [80,81,82]. Due to its antibacterial properties, quaternary ammonium chitosan can be used in anti-inflammatory drugs or as a filler fiber in materials for dressing wounds [82,83].

#### 2.1.3. Formation of Hydrophilic Group

##### Chitosan Esterification Reaction

The esterification reaction of chitosan is the reaction of chitosan with carboxylic acid or an oxy-containing mineral acid. The common acids of chitosan esterification are sulfuric acid, phosphoric acid, and chlorosulfonic acid [84,85]. Sulfated chitosan has a significant anticoagulant activity due to its structural similarity to heparin, and it can be used as an alternative to anticoagulants [86].

##### Chitosan Etherification Modification

The hydroxyl group in the chitosan molecule reacts with a alkylating reagent agent (e.g., dimethyl sulfate, chloroacetic acid, and ethylene oxide) to form a chitosan-etherified derivative. Chitosan-etherified derivatives are soluble in water and have the dual structure and function of high molecular chitosan and low molecular ether [87]. The etherified product has good moisture retention, bacteriostasis, and non-toxicity, and it can be used in medical materials and pharmaceutical fields [88].

### 2.2. Improving Chitosan Mucoadhesion

Mucosal adhesion is the ability of a material to adhere to the mucosa and provide temporary retention. Polymers with mucosal adhesion typically have strong hydrogen bond groups (carboxyl, hydroxyl, amino, and sulfate groups) or strong anion/cation charges, such as chitosan and its derivatives [89]. Chitosan achieves a mucosal adsorption effect through electrostatic attraction with mucosal proteins, along with hydrogen bonding and the material’s hydrophobic properties [90]. Chitosan derivatives generally achieve mucosal adhesion through hydrogen bonding or non-specific, non-covalent, and electrostatic interactions [91]. Strong hydrogen bonding groups can also be introduced to improve mucosal adhesion, such as carboxylated chitosan [89]. Thiolated chitosan derivatives enhance mucosal adhesion through the formation of covalent bonds between free thiol groups and cysteine-containing glycoproteins in mucus [91]. Thiolated chitosan derivatives prepared by the annealing method have stronger adhesion, hydration ability, and drug release properties than thiolated chitosan derivatives that are prepared by other methods [92]. Compared with pure drugs, the amount of insulin that is released by thiolated chitosan xerogels has been shown to increase by 1.7 times. Consequently, thiolated chitosan derivatives can be used in oral drug delivery systems [93,94]. Enhancing the charge of chitosan can improve mucosal adhesion, such as in the case of quaternary ammonium chitosan [95,96]. Here, ovalbumin was conjugated with N-trimethylaminoethylmethacrylate chitosan. After intranasal administration, the conjugated polymer significantly increased the amount of ovalbumin that was absorbed by mouse mononuclear macrophages and improved the efficiency of transporting ovalbumin to deep neck lymph nodes. After three nasal immunizations with a conjugate, a strong systemic and mucosal immune response in mice was induced. Quaternary ammonium chitosan derivatives can be administered via the nasal cavity to prevent respiratory infection diseases [97]. The mucosal adhesion of chitosan derivatives has broad application prospects in both oral and respiratory drug delivery systems.

### 2.3. Improving of Chitosan pH Sensitivity

A pH-sensitive material is a type of material that changes its volume or shape as the pH of its environment changes [98,99]. A change in the intramolecular or intermolecular force of the polymer can be produced, depending on the change in pH. Here, this is manifested as a macroscopic change in the properties of the polymer. A pH-sensitive material can achieve the controlled release of a drug in a delivery system based on continuous changes in the pH of the gastrointestinal tract in the body. Targeted administration can be achieved according to the difference in pH between the lesion and the normal physiological state of the body [100].

pH sensitivity can be increased by introducing a sensitive acyl group in chitosan. A hydrogel with good pH sensitivity was prepared by an alginate and graft copolymer of methoxypolyethylene glycol and carboxymethyl chitosan (mPEG-g-CMC) [101]. Hydrogel materials are sensitive to pH reactions. A pH-sensitive hydrogel that was obtained by crosslinking chitosan with polyacrylic acid contained amoxicillin and meloxicam, and its release rate increased with increase of pH [102]. The methacrylic chitosan hydrogel swelled at a pH < 5, while it shrunk at a pH ≥ 7.4. Cytocompatibility studies were performed with NIH/3T3 fibroblasts (embryonic fibroblasts); cell proliferation or adhesion was suppressed when seeded on hydrogel surfaces compared to tissue culture plastic, but no measurable cell death was observed. It can be seen that the methacrylic chitosan hydrogel is not toxic to fibroblasts, showing specific wound healing stages and accelerated pH-dependent wound healing [103]. Bovine serum albumin (BSA), as a protein drug, was encapsulated in a hydrogel. Here, the results showed that the release rate was lower at a pH of 1.2 and was increased at a pH of 7.4 [101]. A carboxymethyl chitosan/alginate (PECs) hydrogel showed significant pH sensitivity, and the cumulative release amount of a protein at a pH of 7.4 was higher than at a pH of 1.2. Therefore, PECs hydrogels can deliver proteins to the intestine for targeted administration [104].

### 2.4. Targeting Modification

The targeted delivery of drugs is critical to improving treatment outcomes and reducing side effects. There are currently many ways to deliver drugs to specific sites of action. Chitosan derivatives have been driving the development of safe and effective drug delivery systems due to their unique physicochemical and biological properties [105,106].

#### 2.4.1. Colon-Specific Delivery

Colon-specific drug delivery systems have been paid increasing attention for the treatment of diseases such as Crohn’s disease, ulcerative colitis, and irritable bowel syndrome. The main obstacle to delivery here is absorption and degradation via the upper gastrointestinal tract. Therefore, elucidating how to prevent the degradation of drugs in the stomach and small intestine is the research direction of colon-specific drug delivery systems [107,108].

5-fluorouracil (5-FU) is a hydrophilic drug that has been widely used for the treatment of colorectal cancer. The water-soluble amphoteric chitosan derivative (CTAA) is formed by using trimellitic acid chloride, where CTAA is crosslinked with alginate to prepare a film and the CTAA/alginate film can protect 5-FU from being absorbed and degraded by the upper gastrointestinal tract before reaching the colon [109]. O-carboxymethyl chitosan (OCMC) is a carboxymethylated derivative. When compared with other natural forms, OCMC has a better water solubility and more desirable pH sensitivity, and, thus, it can be used as a carrier for intestine-targeted drug delivery. Spherical microcapsules (GA-OCMC LbL) with a core-shell structure were prepared by a layer-by-layer assembly (LbL) with the use of gum arabic (GA) and OCMC. Pharmacokinetic analysis showed that the GA-OCMC LbL not only improved the bioavailability of omeprazole, it also enhanced stability in simulated gastric fluid, indicating that the GA-OCMC LbL is a promising vector for intestine-targeted delivery [110].

#### 2.4.2. Liver-Targeted Delivery

Typically, liver targeting systems employ reticulated endothelial passive capture microparticles or active targeting based on recognition between liver receptors and ligand-bearing microparticles [111]. Fatty-acid-modified quaternary ammonium chitosan derivative nanoparticles were used as a carrier to deliver insulin into the liver, and the results showed that the nanoparticles had a higher hepatocyte uptake and a better anti-diabetic efficacy [80].

Ferulic acid is a promising antioxidant drug that can treat liver cancer. Glycyrrhizic acid and modified chitosan nanoparticles have been used for the liver-targeted delivery of ferulic acid. Here, the results showed a cell viability of 70.6% at a concentration of 300 μg/mL, indicating that the nanoparticles had biocompatibility and non-toxicity. The release of ferulic acid from chitosan nanoparticles loaded with ferulic acid reached 13.34% of the total injected dose after 6 h, showing that the nanoparticles could effectively deliver ferulic acid to the liver [111].

#### 2.4.3. Kidney and Lung Targeted Delivery

Proximal tubular cells and mesenchymal fibroblasts are major targets for the delivery of renal drugs, as they play a key role in many kidney diseases. A carrier that has been successfully used for targeted delivery to the kidneys is acetylated low molecular weight chitosan (LMWC). Here, researchers coupled prednisone with LMWC (19 kDa), and the distribution of prednisone in the kidney was 13 times higher than that of the prednisone alone, which showed that the LMWC could serve as a renal targeting delivery carrier [112]. LMWC may be specifically taken up by tubular cells by megalin, and it can be cleared from the kidneys faster than the lysozyme [113].

A novel folic acid-grafted polyethylene glycol chitosan copolymer (F-PEG-HTCC) was synthesized, and F-PEG-HTCC nanoparticles loaded with Taxol were prepared. The results showed that the nanoparticles had good pharmacokinetic characteristics after reaching the lungs, where the distribution of paclitaxel was limited to the lungs for 6 h; this showed that the nanoparticle delivery method can effectively reduce the side effects of highly toxic drugs [114].

## 3. Application of Chitosan Derivatives as Biomedical Materials

### 3.1. Application in the Antibacterial Materials

Antibacterial materials refer to a new class of functional materials that have the function of killing or inhibiting microbes [115]. There are many substances in nature that have good bactericidal or microbicidal activities [116,117,118]; however, antibacterial materials are a kind of new functional material that have the ability to inhibit or kill bacteria through the addition of certain antibacterial substances, such as antibacterial plastics, antibacterial fibers and fabrics, antibacterial ceramics, and antibacterial metal materials [119,120,121,122]. Chitosan and chitosan derivatives have been widely used as non-toxic or low-toxicity antibacterial materials [123]. Among them, chitosan quaternary ammonium salt is the most widely used [15,83]. Table 1 summarizes the antimicrobial species and applications of quaternized chitosan derivatives.

Compared with chitosan, quaternized chitosan has a significantly increased antibacterial activity and can be used in anti-inflammatory drugs or as a filler fiber in materials for dressing wounds [82,83]. The antibacterial mechanism of chitosan and quaternized chitosan is still inconclusive. There are only three speculations [15,81]: (1) Chitosan and chitosan derivatives are positively charged, while bacteria are negatively charged, causing them to attract and interact with each other due to electrostatic adsorption; (2) after adsorbing bacteria, chitosan and chitosan derivatives enter the inside of bacterial cells and bind to DNA, which interferes with the transcription of bacterial DNA, thereby inhibiting the growth of bacteria; (3) chitosan and chitosan derivatives inhibit the uptake of trace elements and nutrients that are necessary for cell growth. The antibacterial mechanism of TMC is to form a complex with the cell membrane or interfere with gene expression to achieve an antibacterial effect [130]. Cationic molecules are the active site of the polymer. Therefore, with an increase in positive charge, the antibacterial activity of TMC is enhanced. The antibacterial activity of TMC decreases with the decrease of pH under acidic conditions, and the antibacterial activity is lower under weak alkaline conditions than acidic conditions [72,75,131].

### 3.2. Bone Tissue Engineering Material

Bone tissue engineering (BTE) refers to the high-concentration osteoblasts, bone marrow stromal stem cells or chondrocytes that are isolated from the organism itself and which are cultured in vitro and transplanted into a cell scaffold [132,133,134]. A perfect scaffold must be biodegradable and biocompatible, promote cell adhesion and proliferation, and preserve cell metabolism. An implantable stent must have a high degree of compatibility with the body, with suitable mechanical properties, morphology, porosity, healing, and tissue replacement capabilities [1].

A bone graft or stent should mimic the structure and properties of the natural bone extracellular matrix (ECM) and provide all the necessary environmental conditions in the natural bone, which is an area of BTE that needs to be addressed. Current synthetic bone tissue engineering materials consist essentially of hydroxyapatite, protein, and polysaccharides [135,136]. Table 2 shows the applications of common chitosan derivatives in bone tissue engineering.

CMCS is a commonly used in bone tissue engineering [137,138,139,140,141]. In addition to the applications shown in Table 2, CMCS can also be used to make nanofiber scaffolds [144]. In BTE, TMC and heparinoid are commonly used materials for the preparation of periosteal mimics. Almodovar et al. developed an LbL assembly of polyelectrolyte complexes by using TMC as a polycation and heparin as a polyanion, and the LbL assembly was used as a periosteal mimetic to provide osteoprogenitor cells and improve bone and allograft compatibility [145]. Hydroxyalkyl chitosan derivatives are also used in BTE; however, only hydroxypropyl chitosan (HPCS), hydroxybutyl chitosan (HBCS), and hydroxyethyl chitosan (HECS) are currently in use for bone tissue engineering [146,147,148].

## 4. Application of Chitosan Derivative Nanoparticles in Drug Delivery

A drug delivery system is a technical system that comprehensively regulates the distribution of drugs in a living body in terms of delivery space, time, and dosage [149]. The goal is to deliver the right amount of the drug to the right place at the right time, increasing drug bioavailability and reducing costs and side effects [150]. A drug delivery system is a fusion of medicine, engineering (materials, mechanics, and electronics), and pharmacy. The objectives of research here include the drug itself, the drug carrier, and the related delivery technology, as well as the physical and chemical modification of the drug or carrier [151,152]. Chitosan derivative nanoparticles have better bioadhesion and permeability, which can improve the delivery and transport of drugs. Chitosan derivative nanoparticles have important applications in targeting, sustained release, and increasing drug absorption. At present, chitosan derivative nanoparticles are mainly used for sustained release, the preparation of targeted drugs, and as vectors for gene therapy.

### 4.1. Delivery Carrier

Chitosan and its derivatives are mainly found as microspheres, nanoparticles, micelles, and gels in delivery carriers [153,154,155]. The particle sizes of these microspheres are generally in the range of 1–500 μm, and the particle sizes of the nanoparticles are smaller than the particle sizes of the microspheres and are within 100 nm. The small size of nanoparticles enables them to pass through various biological barriers in order to deliver drugs to target sites [156]. A micelle is a core-shell structural material with good stability, tissue permeability, and sustained drug release properties [157]. Amphiphilic chitosan, with a self-assembling micelle, can improve the solubility, biological activity, and targeted delivery of fat-soluble drugs [158]. A gel is a material that has a three-dimensional spatial polymerization capability and is capable of accommodating a large amount of water in its slightly crosslinked network structure. Gels have many adjustable properties, including their flexibility and deformability, dispersibility in biological fluids, controlled stability, biodegradability, and chemical function [159,160]. Compared with nanoparticles, gels have better mucoadhesivity and permeability and can transport small molecules, all of which gives them great potential in biomedical fields [161].

### 4.2. Controlled Drug Delivery

Drug control and sustained release are promising areas of research [162]. Some drugs have a very short release time and are rapidly consumed, resulting in a decrease in plasma levels. Therefore, more doses of the drug are needed to maintain plasma balance, causing discomfort to the patient. Scientists are working hard to develop novel drug delivery systems in order to provide appropriate drug concentrations to meet therapeutic needs [163]. The ideal drug release is rapid, at a constant rate, and sustained so that the drug be immediately effective while achieving a long effect [164]. Chitosan derivative nanoparticles can easily achieve a sustained slow release, increasing bioavailability and therapeutic efficacy while reducing side effects [165].

Proteins are the drugs of choice for the treatment of various diseases due to their targeting and good biocompatibility. However, protein drugs also have some drawbacks [166]. For example, proteins are easily degraded by enzymes, have low permeability in the intestinal epithelium, and have poor oral absorption. These factors limit the applications of protein drugs [167]. In recent years, chitosan derivative nanoparticles have received considerable attention for use as protein drug delivery vehicles. These nanoparticles have received considerable attention and can be used to deliver proteins.

#### 4.2.1. Chitosan Derivative Nanoparticles for the Delivery of Polypeptide

Chitosan derivative nanoparticles interact with peptides through strong hydrogen bonds and static electricity, obtaining peptide-loaded nanoparticles. Nanoparticle-loaded peptides have a better thermal stability and are controllable via in vitro release when compared to free peptides [168]. Fatty acid-modified quaternary ammonium chitosan nanoparticles loaded with insulin have been shown to have an encapsulation efficiency and loading capacity of over 98%, as nanoparticle-loaded insulin is more efficient than a free insulin group [80]. The oral delivery of insulin needs to overcome the barrier of gastrointestinal tract digestion and absorption [169]. Fucoidan (FD) has hypoglycemic effects. Nanoparticles (NPs) prepared by TMC and FD were loaded with insulin. Here, the TMC/FD NPs were pH sensitive and could protect insulin from degradation in the gastrointestinal tract, and TMC/FD NPs enhanced the cellular transport of insulin across the intestinal barrier [170]. The delivery of insulin by glycerol monocaprylate-modified chitosan nanoparticles has also achieved the same effects as delivery via TMC/FD NPs [171].

#### 4.2.2. Chitosan Derivative Nanoparticles for the Delivery of Gene

Gene therapy is a promising strategy for challenging diseases. A key step in gene therapy is the successful delivery of genes [172]. Therefore, a safe and effective gene delivery system is critical for the successful application of gene therapy [173]. The delivery carriers of genes include viral and non-viral vectors. Compared with non-viral vectors, the transfection rate and immunogenicity of the viral vector are both higher, but the vector is more toxic, the capacity of the target gene is smaller, the targeting specificity is worse, the preparation is more complicated, and the cost is higher. Compared with viral vectors, non-viral vectors feature an ease of production, high yield, and low cost, and they can be widely used for drug delivery [174,175,176,177,178]. Chitosan derivative nanoparticles, as non-viral vectors, have excellent solubility, biodegradability, biocompatibility, non-toxicity, and a higher transfection rate than chitosan nanoparticles [73,98,179]. Table 3 shows a gene delivery carrier-containing a chitosan derivative and its applications.

Genes can regulate signaling networks and promote tumor suppression through chemotherapy, which is important for the treatment of tumors [189]. However, naked nucleic acids cannot cross cell membranes and are easily degraded by nucleases [190]. TMC can be further modified to protect genes from degradation by nucleases in serum [191,192,193]. For example, methoxy polyethylene glycol-modified trimethyl chitosan (mPEG-TMC) has been covalently linked to doxorubicin (DOX) and cis-itaconic anhydride (CA) in order to give mPEG-TCD NPs, where anti-tumor effects have shown that the mPEG-TCD NPs exhibit better anti-tumor activities when compared with DOX and plasmid DNA alone [194,195]. O-carboxymethyl chitosan nanoparticles have been shown to have the ability to inhibit tumor cell migration in vitro [7]. The poly-β-amino ester nanoparticle loading gene, after the addition of thiolated O-carboxymethyl chitosan, showed a higher cell transfection rate, as the loaded genes of the poly-β-amino ester and thiolated O-carboxymethyl chitosan composite nanoparticles had higher cell transfection rates than the nanoparticles of the former alone [184]. The target ligand can also be used to modify chitosan derivatives in order to improve the targeted delivery of genes. Target ligands have been shown to improve tumor-specific delivery, promote cellular uptake, and reduce side effects [196].

## 5. Applications of Chitosan Derivative Nanoparticles in Mucosal Immunity

The body mainly relies on immune response to destroy and repel pathogenic microorganisms that enter the body for the prevention of infectious diseases. There are three immune defense systems in human and animal bodies, with mucosal immunity being the first defense system against infection. The mucosal immune system refers to lymphoid tissues that are widely distributed in the respiratory tract, gastrointestinal tract, genitourinary mucosa, and some exocrine glands, and this system is the main site for performing local specific immune function [197,198].

**Figure 6:**
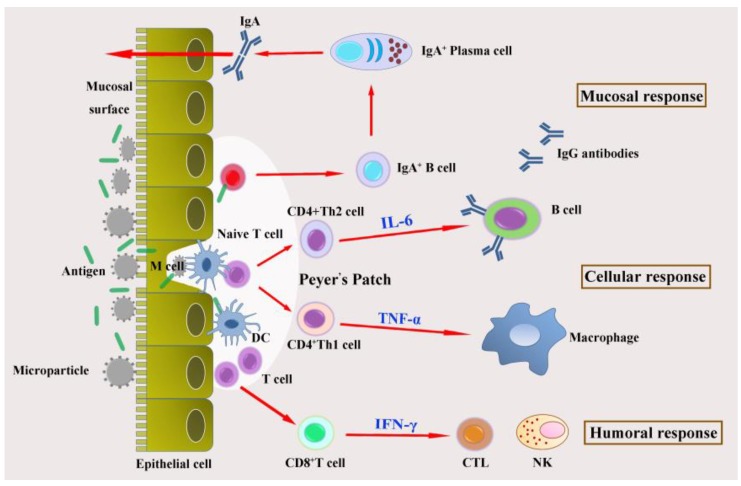
Immunity causes antibody production to be immune.

Nowadays, vaccination is one of the most effective and economical strategies for humans and animals to control and prevent the spread of infectious diseases. It is worth noting that, in recent years, with the advancement of immunology, biotechnology, and nanotechnology, etc., some intractable cancers have been included in the scope of vaccination targets, and great therapeutic effects in some patients have been achieved [199,200]. Traditionally, vaccines are mainly delivered by intramuscular injection, but this route generally cannot induce effective mucosal immunity. Mucosal vaccines have some advantages compared with traditionally vaccines, and most infections occur at a mucosal surface or spread from mucosal surfaces [201]. Studies have shown that mucosal administration could provide an early defense against invading pathogens, as the local antibodies of the mucosa work faster than serum antibodies, are higher in level, and have a greater maintenance time [202,203]. The process of immunity is shown in Figure 6 [204].

Chitosan and chitosan derivatives can open tight junctions between epithelial cells to facilitate the transmembranal delivery of drugs. Compared with chitosan nanoparticles, chitosan derivative nanoparticles have a better mucoadhesivity, higher water solubility, and promote the better absorption of antigens; thus, chitosan derivative nanoparticles can serve as vaccine adjuvants or delivery carriers in the case of mucosal immunity [204]. In our previous study, our group prepared a Newcastle disease live vaccine/*O*-2′-Hydroxypropyl trimethyl ammonium chloride chitosan nanoparticles, where the results showed that the chickens that were intranasally immunized with the nanoparticles had stronger cellular, humoral, and mucosal immunity than those intramuscularly immunized with the live attenuated Newcastle disease vaccine, indicating that *O*-2′-Hydroxypropyl trimethyl ammonium chloride chitosan nanoparticles can be used as vaccine adjuvants and delivery carriers in the case of mucosal immunity [205].

To achieve a higher mucosal immune effect, composite biological nanomaterials were synthesized [206,207]. Reshma et al. prepared a peptide vaccine by binding TMC to a peptide antigen and polyglutamic acid (PGA), and the vaccine induced higher levels of mucosal antibodies compared with the mucosal adjuvant cholera toxin B subunit [208]. N-2-HACC and CMCS were synthesized as vaccine adjuvants and delivery systems in our group, where an animal experiment showed that the intranasal N-2-HACC/CMCS nanoparticles produced higher IgG (immunoglobulin G) and sIgA (secretory immunoglobulin A) antibody titers and higher levels of cytokines than the commercially available vaccine did [209]. Additionally, our team also synthesized chitosan-coated PLGA(poly(lactic-co-glycolic acid) nanoparticles, and NDV (Newcastle disease virus) energy-containing DNA that was encapsulated in the nanoparticles was prepared to evaluate the mucosal immune response; the results indicated that the nanoparticles induced stronger cellular, humoral, and mucosal immune responses than the plasmid DNA alone, showing that the chitosan-coated PLGA nanoparticles could be used as an efficient delivery system for mucosal immunization in the case of DNA vaccines [210].

## 6. Prospects

As a kind of biodegradable polymer material with excellent performance, chitosan is widely used in medical materials and biomedicines. In order to improve the water solubility of chitosan and broaden the scope and fields of its applications, chitosan derivatives with excellent properties, such as hydrophilicity, pH sensitivity, and targeting, have been synthesized through chemical reactions. Chitosan derivatives are promising drug excipients. A drug-loading system, including micelles, nanoparticles, microspheres, and hydrogels, that is prepared by chitosan derivatives, can increase the stability of drugs and release drugs in a sustained and slow manner. Thus, chitosan derivative delivery systems for drugs or vaccines can reduce side effects and improve the bioavailability of drugs, which has increased interest for chitosan derivatives in the field of biomedicine.

In order to improve current drug delivery systems, it is necessary to study the properties of drugs and new carrier materials. It is important to study new biodegradable materials that are non-toxic to humans and the environment that can overcome the shortcomings of specific drugs. The preparation of multifunctional composite nanoparticles is required to prevent the effects of drugs from adverse conditions and to prolong the release of drugs at the target site. Among the existing polymers, chitosan derivatives seem to be more attractive and can be used for the controlled release of drugs, and their modification with new solvents can be converted into drug delivery systems, especially for the controlled release and persistence of the released drugs. With additional research, it is believed that the physical and chemical properties of chitosan derivative nanomaterials can be continuously improved by chemical modification methods and can also be made to be more suitable for use in medical materials and drug delivery systems. Chitosan derivatives will have broader prospects in biomedicine in the future.

## Figures and Tables

**Table 1:** Applications of quaternary ammonium chitosan in antibacterial.

Name	Antibacterial Species	Application
Ammonium N-alkyl chitosan particles	*Staphylococcus aureus*, *Escherichia coli*	Biomedical devices, textile industry [124]
Quaternized N-alkyl chitosan film	*Staphylococcus aureus*, *Escherichia coli*	Antibacterial material [125]
Quaternized N-aryl chitosan	*Staphylococcus aureus*	Antibacterial material [126]
Quaternary ammonium chitosan-containing monosaccharide or disaccharide moiety	*Staphylococcus aureus*, *Escherichia coli*	Antibacterial agents [127]
O-imidazolyl quaternary ammonium chitosan	*Botrytis cinerea*	Anti-cancer, anti-virus, anti-diabetes, enzyme inhibition and anti-tuberculosis [128]
Trimethyl ammonium chitosan	*Aspergillus flavus*	Biodegradable fungicide [129]
Glutaraldehyde cross-linked chitosan quaternary ammonium salt film	*Escherichia coli*, *Staphylococcus aureus*, *Pseudomonas aeruginosa*	Antibacterial coating [81]

**Table 2:** Applications of common chitosan derivatives in bone tissue engineering.

Chitosan Derivative	Complex	Application	Attributes
Carboxymethyl chitosan	Fibroin/CMCS/strontium replaces hydroxyapatite/cellulose nanocrystals	Preparation bracket	Improving adhesion and proliferation of osteoblasts [137]
	Tyrosinase/CMCS/gelatin/nano hydroxyapatite	Treatment of irregular small bone defects	Injectable gel to promote osteoblast differentiation and maturation [138]
	Silk fibroin/CMCS/vitamin C	Preparation bracket	Enhanced cell proliferation, proliferation, and alkaline phosphatase activity, promoting calcium phosphate deposition in the scaffold [53]
	CMCS/nano hydroxyapatite/graphene oxide	Preparation bracket	Enhances osteoinductivity and promotes new bone formation [139]
	Gelatin/CMCS/LAPONITE	Preparation bracket	Enhances mechanical properties and promotes bone marrow stem cell attachment, proliferation and osteogenic differentiation [140]
Carboxymethyl chitosan nanofiber	Hydroxyapatite coated electrospun CMCS nanofibers	Preparation bracket	Enhances ALP activity and promotes osteoblast differentiation and maturation [141]
N, O-carboxymethyl chitosan	Hydroxyapatite/N, O-carboxymethyl chitosan/fucoidan	Preparation bracket	Increased pore size and mechanical properties promote osteoblast differentiation [61]
Trimethyl chitosan	N,N,N-trimethyl chitosan-heparin polyelectrolyte multilayer	Bionic periosteum	Good cell compatibility and support osteoblast differentiation [142]
Hydroxypropyltrimethylammonium chloride chitosan	Alginate/HACC/oyster shell powder	Preparation bracket	Improve mechanical properties and enhance stent surface area [143]

**Table 3:** Gene carrier-containing chitosan derivative and applications.

Carrier	Drug	Application	Main Findings/Features
Polyethyleneimine/chitosan-4-thio-butyl-oxime	siRNA	Colorectal cancer	Increased transfection rate [180]
Polyethylene glycol/O-carboxymethyl chitosan/low molecular weight polyethylene imine	siRNA	Breast cancer	Enhanced targeting and cell transfection rates [181]
Methyl methacrylate-modified chitosan	Curcumin	Mammalian cancer cell line (A549, HeLa, HepG2)	Increased transfection rate [182]
Ethylene glycol chitosan–dequalinium nanoparticles	Curcumin	Tumor treatment	Targeted delivery [183]
Poly-β-amino ester/thiolated O-carboxymethyl chitosan nanoparticles	RNAi	Lung cancer	Increased transfection rate and increased cellular uptake [184]
Aminotetrazole functionalized magnetic chitosan nanocomposites	Plasmid DNA	HEK-293T cell line	Increased transfection rate [185]
Polyethylene glycol grafted chitosan nanoparticles	p53	Tumor cell	High transfection efficiency and increased cellular uptake [186]
Chitosan derivative-modified mesoporous silica microspheres	DOX, p53	Tumor treatment	Increased transfection rate, sustained release [187]
Trans transcriptional activator/poly(N-3-benzyloxycarbonyl-lysine) chitosan	p53, DOX	Cancer treatment	Improve transfection efficiency and drug delivery efficiency [188]
